# CT texture analysis: a potential tool for prediction of survival in patients with metastatic clear cell carcinoma treated with sunitinib

**DOI:** 10.1186/s40644-017-0106-8

**Published:** 2017-01-23

**Authors:** Masoom A. Haider, Alireza Vosough, Farzad Khalvati, Alexander Kiss, Balaji Ganeshan, Georg A. Bjarnason

**Affiliations:** 1 Department of Medical Imaging, Sunnybrook Health Sciences Center, University of Toronto, Rm AG-46, 2075 Bayview Ave, Toronto, Onatrio M4N 3M5 Canada; 2 Department of Radiology, North Bristol NHS Trust, Southmead Hospital, Bristol, UK; 3 Sunnybrook Health Sciences Center, Toronto, Canada; 4 Institute of Nuclear Medicine, University College London, London, UK; 5 Sunnybrook Odette Cancer Centre, Division of medical Oncology, University of Toronto, Toronto, ON Canada

**Keywords:** Prediction of outcome, Metastatic clear cell carcinoma, Quantitative imaging biomarkers, CT image features, CT texture analysis

## Abstract

**Background:**

To assess CT texture based quantitative imaging biomarkers in the prediction of progression free survival (PFS) and overall survival (OS) in patients with clear cell renal cell carcinoma undergoing treatment with Sunitinib.

**Methods:**

In this retrospective study, measurable lesions of 40 patients were selected based on RECIST criteria on standard contrast enhanced CT before and 2 months after treatment with Sunitinib. CT Texture analysis was performed using TexRAD research software (TexRAD Ltd, Cambridge, UK). Using a Cox regression model, correlation of texture parameters with measured time to progression and overall survival were assessed. Evaluation of combined International Metastatic Renal-Cell Carcinoma Database Consortium Model (IMDC) score with texture parameters was also performed.

**Results:**

Size normalized standard deviation (nSD) alone at baseline and follow-up after treatment was a predictor of OS (Hazard ratio (HR) = 0.01 and 0.02; 95% confidence intervals (CI): 0.00 – 0.29 and 0.00 – 0.39; *p* = 0.01 and 0.01). Entropy following treatment and entropy change before and after treatment were both significant predictors of OS (HR = 2.68 and 87.77; 95% CI = 1.14 – 6.29 and 1.26 – 6115.69; *p* = 0.02 and *p* = 0.04). nSD was also a predictor of PFS at baseline and follow-up (HR = 0.01 and 0.01: 95% CI: 0.00 – 0.31 and 0.001 – 0.22; *p* = 0.01 and *p* = 0.003). When nSD at baseline or at follow-up was combined with IMDC, it improved the association with OS and PFS compared to IMDC alone.

**Conclusion:**

Size normalized standard deviation from CT at baseline and follow-up scans is correlated with OS and PFS in clear cell renal cell carcinoma treated with Sunitinib.

## Background

Multi-targeted tyrosine kinase inhibitor (TKI) therapy with Sunitinib is a standard treatment of metastatic clear cell renal cell carcinoma (RCC). Non-imaging related clinical prognostic factors have been identified for patients receiving targeted therapy and introduced into treatment guidelines and used to stratify patients on clinical trials [1]. This clinical prognostic model and the associated factors are described in the International Metastatic Renal-Cell Carcinoma Database Consortium Model (IMDC), which is used most commonly [2].

It is well known that enhancement features of RCC can change on contrast enhanced CT in patients receiving TKI’s such as Sunitinib and this is not always reflected in an early change in the size of tumors thus limiting the application of RECIST criteria [3]. Multiple alternative response criteria which combine size and enhancement change such as Choi, modified Choi and Morphology Attenuation, Size and Structure (MASS) criteria have demonstrated a predictive ability by combining size and enhancement criteria to predict progression free survival (PFS) in patients with metastatic RCC [4–8].

Intratumoral heterogeneity is a recognized feature of cancer behavior and in particular, therapeutic resistance [9]. Analysis of tumor heterogeneity using CT texture analysis has shown promise as a prognostic and predictive measure in RCC. A previous study by Goh et al. showed that CT texture analysis reflecting tumor heterogeneity is an independent factor associated with PFS and has the potential to be used as a predictive imaging biomarker of response of metastatic RCC of various histologic types [10]. A more recent study has also included a variety of RCC histologies and confirmed potential prognostic value of CT texture features in assessment of the primary tumor site and outcome [11]. There have been other studies on the prognostic value of CT texture features for different types of cancer including breast [12] lung [13], hepatic metastatic colorectal cancer [14], pancreatic cancer [15] as well as reproducibility of CT texture parameters [16]. To our knowledge, a study specifically reviewing the potential prognostic and predictive value of texture features in a pure clear cell RCC cohort and evaluating this in the light of the IMDC prognostic score has not been performed.

The purpose of this study was to assess CT texture analysis based Quantitative Imaging Biomarkers (QIB’s) in the prediction of PFS and Overall Survival (OS) in patients with clear cell RCC undergoing treatment with Sunitinib.

## Methods

### Patients

The institutional research ethics board approved this retrospective single institution study and waived the requirement for informed consent.

Patients with metastatic clear cell carcinoma who received the TKI Sunitinib as first or second line therapy at our institution between December 2005 and March 2010 were identified from institutional renal cancer database. An attempt was made to optimize the activity of Sunitinib by treating each patient to toxicity using individualized dose and schedule [17]. An IMDC prognostic score was assigned to each patient at baseline.

Patients were included if they were TKI naïve and had received Sunitinib as the first or second line treatment for metastatic clear cell carcinoma. Patients were excluded if: their baseline contrast enhanced CT was not performed within 6 weeks before the start of treatment; both their baseline and followup CT were not performed with contrast enhancement; they did not have measurable disease at baseline as defined by RECIST 1.1.

From a total of 172 patients who were identified in the institution database, 132 patients were excluded with 40 patients left for analysis. The most common reasons for exclusion was lack of availability for the pre-treatment or first follow-up scan, typically due to imaging being performed at another institution.

### CT examination

All patients underwent contrast enhanced CT examination of their chest, abdomen and pelvis (GE Lightspeed Plus or GE Lightspeed VCT) following injection of 100 ml of an iodinated contrast agent (Omnipaque 300, Iohexol, GE Healthcare, Princeton, NJ, USA) at a rate of 3 ml/s via an automated injector.

Images of the thorax were analyzed in arterial phase (25-s delay) and images of the abdomen and pelvis were analyzed in portal venous phase (70-s delay) with the following acquisition parameters: 120 kV; auto mA and Smart mA (angular and z-axis modulation); pitch 0.75:1 and 0.9:1; 20 mm collimation, 5 mm slice thickness and 40 mm collimation reconstructed; scan field of view (FOV) 50 cm and display FOV adjusted to patient size; matrix: 512x512 (pixel spacing: 0.933 mm). Region of interest (ROI) was drawn by a radiologist with 6 years of experience of reporting abdominal CT who was blinded to the clinical outcome. Each ROI was drawn on the slice through the largest diameter of the tumor site.

### CT Texture Analysis

Target lesions were selected according to RECIST, version 1.1 (maximum of five target lesions, maximum of two lesions per organ). If the patient had not had resection of primary tumor, it was included as a measurable tumor and used for analysis. RECIST 1.1 criteria were used to identify disease progression, which was confirmed using subsequent imaging. Using RECIST 1.1, initial response after two cycles of treatment was evaluated. CT texture analysis of the lesions was performed using TexRAD commercial research software (TexRAD Ltd, www.texrad.com, part of Feedback Plc, Cambridge, UK) by drawing a ROI around the peripheral margin of the selected lesions used for RECIST. Metastases/primary lesions less than 1 cm in maximal diameter were not included in the analysis. Air, streak artifacts and dense calcifications were excluded from the regions of interest. CT texture analysis comprised a filtration-histogram technique where the filtration step extracted and enhanced (amplified) features using a band-pass Laplacian of Gaussian spatial scale filter [18]. Quantification was done using different histogram based statistical parameters in the selected region of interest in CT images after the application of the band-pass filter at intermediate scale which was chosen to mimic the scale used by Goh et al. [10].

Histogram based statistical parameters comprised of mean positive pixel intensity (the average value of the positive pixels within the ROI), standard deviation (SD), skewness (symmetry of the pixel intensity distribution), kurtosis (pointiness of the pixel intensity distribution), and entropy which represents irregularity or complexity of pixel intensity in space. These are all first-order statistical features except for entropy which is a second-order statistical feature. A newer secondary histogram parameter derived from the above parameters, size normalized standard deviation (nSD), was also evaluated. This was done as it is known that SD estimate can be affected by the size (meaning the number of pixels) specifically in case of small tumors which may need correction or normalization [18] (Eq. 1).

nSD of a given ROI is calculated as follows:

1 $$ nSD=\frac{Ln(SD)}{Ln(N)} $$

where *SD* is the standard deviation of ROI (i.e., tumor) and *N* is the total number of pixels in the ROI.

These parameters were recorded for each lesion in baseline CT images and the first follow-up CT images after treatment with Sunitinib. Percentage change (*PerC*) of the CT texture parameters before and after treatment were calculated (Eq. 2).

2 $$ PerC=\frac{\left(\mathrm{Parameter}\ \mathrm{p}\mathrm{ost}\ \mathrm{treatment}\right)\ \hbox{--}\ \left(\mathrm{Parameter}\ \mathrm{p}\mathrm{r}\mathrm{e}-\mathrm{treatment}\right)}{\left(\mathrm{Parameter}\ \mathrm{p}\mathrm{r}\mathrm{e} - \mathrm{treatment}\right)}\times 100 $$

As the value of Kurtosis can vary from −3 to +3, to negate division by 0 when calculating the percentage change, we added 3, i.e. [((Kurtosis post treatment +3) - (Kurtosis pre-treatment +3))/(Kurtosis pre-treatment +3)] × 100. In case of Skewness, in order to avoid dividing by 0, only change (not the percentage change) was used, i.e. Skewness post treatment – Skewness pre-treatment.

### Statistical Analysis

For statistical analysis, average texture measurements of all measured lesions in each patient at baseline CT and the first follow-up CT performed about 2 months after the start of treatment, the percentage change from baseline value as well as the percentage change in lesion size based on RECIST 1.1 criteria, which measures the amount of lesion size reduction, were used. PFS was defined as the time from the date of baseline CT to the date of disease progression based on RECIST response criteria. A Cox proportional hazards survival model was performed to determine if any parameter was predictors of OS or PFS. No adjustments for multiple testing were carried out as these analyses are considered exploratory and their results will serve to enhance future larger studies. All statistical analysis was performed using SAS Version 9.3 (SAS Institute, Cary, NC, USA).

The International Metastatic Renal-Cell Carcinoma Database Consortium Model (IMDC) allocates patient to three prognostic groups (good, intermediate and poor) based on the degree of anemia, thrombocytosis, neutrophilia, and hypercalcemia, as well as the Karnofski performance status <80%, and <1 year from diagnosis to treatment. To assess whether any of the CT texture parameters adds to IMDC in prediction of PFS and OS, we ran 2 variable Cox proportional hazards regression models and assessed model fit using −2 log likelihood (−2LL) statistics. For addition of one variable to IMDC, a change of greater than 3.84 (the critical value associated with one degree of freedom for a chi-square statistic) would indicate a significant model improvement. We could not explore models with 3 or more variables due to limitations in sample size. A P value of less than 0.05 was considered to indicate statistical significance.

## Results

The cohort consisted of 35 men and 5 women with a mean age of 60 years (range of 34–76 years). All the patients had clear cell carcinoma. 34 patients received Sunitinib as first line therapy and 6 patients received Sunitinib as the second line treatment after being treated with a non-TKI drug (e.g. interferon). A total of 87 target lesions were analyzed. These lesions were scattered across different locations as listed in Table 1.

**Table 1:** Tumor Sites

Tumor Site	Number of lesions
Lymph node	15
Lung	14
Primary	10
Bone	10
Liver	8
Peritoneum	6
Adrenal	4
Mediastinal Lymph Nodes	4
Nephrectomy bed	4
Other kidney	3
Hilar Lymph Nodes	3
Pleura	1
IVC Thrombus	1
Muscle	1
Spleen	1
Omentum	1
Psoas major muscle	1
Total	87

The baseline contrast enhanced CT of the patients included in the analysis was performed within 6 weeks before the start of treatment (mean was 12.8 day pre-treatment) and their followup CT was performed with contrast enhancement (mean followup time was 67.5 days). Six of 40 patients had progressed during the study period with a PFS of 60 days (range: 64–171 days). The length of follow-up was up to 22.8 months following baseline CT or until death whichever sooner. The significance of each texture parameter in prediction of OS and PFS is summarized in Tables 2 and 3, respectively.

**Table 2:** Imaging parameters as predictors of OS

Variable	*P*-value	Hazard ratio	95% CI
Mean positive pixel intensity prior to treatment	0.34	1.01	0.98 – 1.05
Mean positive pixel intensity following treatment	0.77	1.00	0.99 – 1.02
SD prior to treatment	0.24	0.98	0.96 – 1.01
SD following treatment	0.46	0.99	0.98 – 1.01
Entropy prior to treatment	0.27	1.74	0.65 – 4.68
**Entropy following treatment**	**0.02**	**2.68**	**1.14 – 6.29**
**nSD prior to treatment**	**0.01**	**0.01**	**0.00 – 0.29**
**nSD following treatment**	**0.01**	**0.02**	**0.001 – 0.39**
Kurtosis prior to treatment	0.26	1.24	0.85 – 1.82
Kurtosis following treatment	0.78	1.03	0.81 – 1.32
Skewness prior to treatment	0.15	2.02	0.77 – 5.30
Skewness following treatment	0.30	1.47	0.70 – 3.09
**Percent change in size**	**0.02**	**0.97**	**0.94 – 0.99**
Mean pixel intensity Change	0.37	0.99	0.98 – 1.01
SD Change	0.80	1.14	0.42 – 3.09
**Entropy Change**	**0.04**	**87.77**	**1.26 – 6115.69**
nSD Change	0.39	0.18	0.004 – 8.56
Kurtosis Change	0.46	0.66	0.21 – 2.02
Skewness Change	0.98	0.99	0.60 – 1.65

Entries in bold were significant

*Abbreviations: SD* standard deviation, *nSD* size normalized standard deviation *OS* overall survival

**Table 3:** Imaging parameters as predictors of PFS

Variable	*P*-value	Hazard ratio	95% CI
Mean positive pixel intensity prior to treatment	0.61	1.01	0.98 – 1.04
Mean positive pixel intensity following treatment	0.72	1.00	0.99 – 1.02
SD prior to treatment	0.71	0.99	0.97 – 1.02
SD following treatment	0.33	0.99	0.97 – 1.01
Entropy prior to treatment	0.25	1.84	0.64 – 5.32
Entropy following treatment	0.08	2.23	0.91 – 5.49
**nSD prior to treatment**	**0.01**	**0.01**	**0.000 – 0.31**
**nSD following treatment**	**0.003**	**0.01**	**0.001 – 0.22**
Kurtosis prior to treatment	0.21	1.28	0.87 – 1.87
Kurtosis following treatment	0.34	1.12	0.89 – 1.41
Skewness prior to treatment	0.32	1.66	0.62 – 4.41
Skewness following treatment	0.65	1.20	0.55 – 2.61
Percent change in size	0.14	0.98	0.96 – 1.01
Mean pixel intensity change	0.36	0.99	0.98 – 1.01
SD change	0.51	0.70	0.25 – 2.02
Entropy change	0.21	15.12	0.21 – 1096.9
nSD change	0.06	0.02	0.00 – 1.21
Kurtosis change	0.92	1.06	0.37 – 3.04
Skewness change	0.81	0.94	0.56 – 1.58

Entries in bold were significant

*Abbreviations: SD* standard deviation, *nSD* size normalized standard deviation, *PFS* progression free survival

### Overall survival

Size normalized standard deviation (nSD) prior to treatment with Sunitinib was a significant predictor for OS (*p* = 0.01) such that higher nSD before treatment predicted for increased survival (positive correlation) (HR = 0.01, 95% CI = 0.00 – 0.29). In addition, nSD following treatment with Sunitinib was found to be significant in predicting OS (*p* = 0.01) such that higher nSD after treatment was associated with a lower hazard of death or higher survival (positive correlation) (HR = 0.02, 95% CI = 0.001 – 0.39). Entropy following treatment and entropy change both were significant predictors of OS such that higher entropy following treatment or entropy change predicted decreased survival (negative correlation) (*p* = 0.02, HR = 2.68, 95% CI = 1.14 – 6.29 and *p* = 0.04, HR = 87.77, 95% CI = 1.26 – 6115.69, respectively) (Table 2).

### Progression free survival

With regards to PFS, nSD prior to treatment with Sunitinib was a significant predictor (*p* = 0.01) such that higher nSD before treatment was related to increased PFS (positive correlation) (HR = 0.01, 95% CI = 0.00 – 0.31). Moreover, nSD following treatment with Sunitinib was found to be significant in predicting PFS (*p* = 0.003) such that higher nSD after treatment leads to lower hazard of death or increased PFS (positive correlation) (HR = 0.01, 95% CI = 0.00 – 0.22). Neither entropy following treatment nor entropy change was significant predictor of PFS (Table 3).

The lesion’s size change (percentage change, in terms of reduction, in sum of the lesions’ size in each patient prior to and following treatment with Sunitinib) demonstrated a significant relation to OS (positive correlation) (*p* = 0.02), (HR = 0.97, 95% CI = 0.94 – 1.00), while size change was not a significant predictor of PFS.

### Imaging variables combined with IMDC

To analyze the added predictive value of imaging variables to IMDC, we started with a baseline Cox proportion hazards model that only included the IMDC and then added each of the imaging variables found to be significant in the univariate survival analyses for OS or PFS. The baseline model with IMDC alone was significantly related to OS and had a −2 log likelihood statistic of 181.3. Therefore, to see an improved fit in any of the two-variable models tested, this statistic would have to change by a minimum of 3.84 (a 1° of freedom change for a chi-square statistic). This means that any value of 177.5 or less indicated a significant improvement in model fit. This improvement was seen for all two-variable models tested, however the ones that contained nSD prior to treatment and nSD following treatment showed the greatest improvement in fit. For both of these models, IMDC was no longer significant. Therefore, these variables (i.e., nSD prior to and following treatment) were seen as stronger predictors of survival in a model that included IMDC. The ability of each texture parameter to add to IMDC model in the prediction of OS is summarized in Table 4.

**Table 4:** Imaging variables combined with IMDC to see if they improve on the prediction of OS

Model	IMDC	IMDC Combined Model	Fit statistic value (−2LL)
**Baseline model with IMDC alone**	**Significant**		**181.3**
**IMDC + Entropy following treatment**	**Not Significant**	**Significant**	**176.4**
**IMDC + nSD prior to treatment**	**Not significant**	**Significant**	**176.1**
**IMDC + nSD following treatment**	**Not significant**	**Significant**	**175.3**
IMDC + percent change in lesion size	Not significant	Not significant	177.8
IMDC + Entropy change	Not significant	Not significant	177.4

Entries in bold were significant

*Abbreviations: nSD* size normalized standard deviation, *IMDC* International Metastatic Renal-Cell Carcinoma Database Consortium Model, *OS* overall survival

For PFS models, the baseline model with IMDC alone was not significantly related to PFS and had a −2 log likelihood statistic of 174.4. Therefore to see an improved fit in any of the two-variable models tested, this statistic would have to change by a minimum of 3.84 (a 1° of freedom change for a chi-square statistic). This means that any value of 170.6 or less indicated a significant improvement in model fit. This improvement was only seen for the 2 variable models that contained nSD prior to treatment and nSD following treatment. For both of these models, the nSD variables significantly improved the model in predicting PFS whereas in neither model IMDC was significant. Therefore, these variables were seen as stronger predictors of survival in a model that included IMDC. The ability of each texture parameter to improve IMDC model in the prediction of PFS is summarized in Table 5.

**Table 5:** Imaging variables combined with IMDC to see if they improve on prediction of the PFS

Model	IMDC	IMDC Combined Model	Fit statistic value (−2LL)
Baseline model with IMDC alone	Not significant		174.4
**IMDC + nSD pre treatment**	**Not significant**	**Significant**	**169.3**
**IMDC + nSD post treatment**	**Not significant**	**Significant**	**165.9**

Entries in bold were significant

*Abbreviations: nSD* = size normalized standard deviation, *IMDC* International Metastatic Renal-Cell Carcinoma Database Consortium Model, *PFS* progression free survival

When added, the lesion size (percentage change in sum of the largest diameter of the lesions’ size prior to and post treatment) did not make a significant improvement to IMDC in prediction of OS. Finally, separate univariate Cox proportional hazards models were run, one for percent change in size, the other for nSD prior to treatment, to compare the two variables. nSD prior to treatment was found to be a better predictor of OS (*p* = 0.01 versus 0.02) with HR = 0.01, 95% CI = 0.00 – 0.29).

**Fig. 1:**
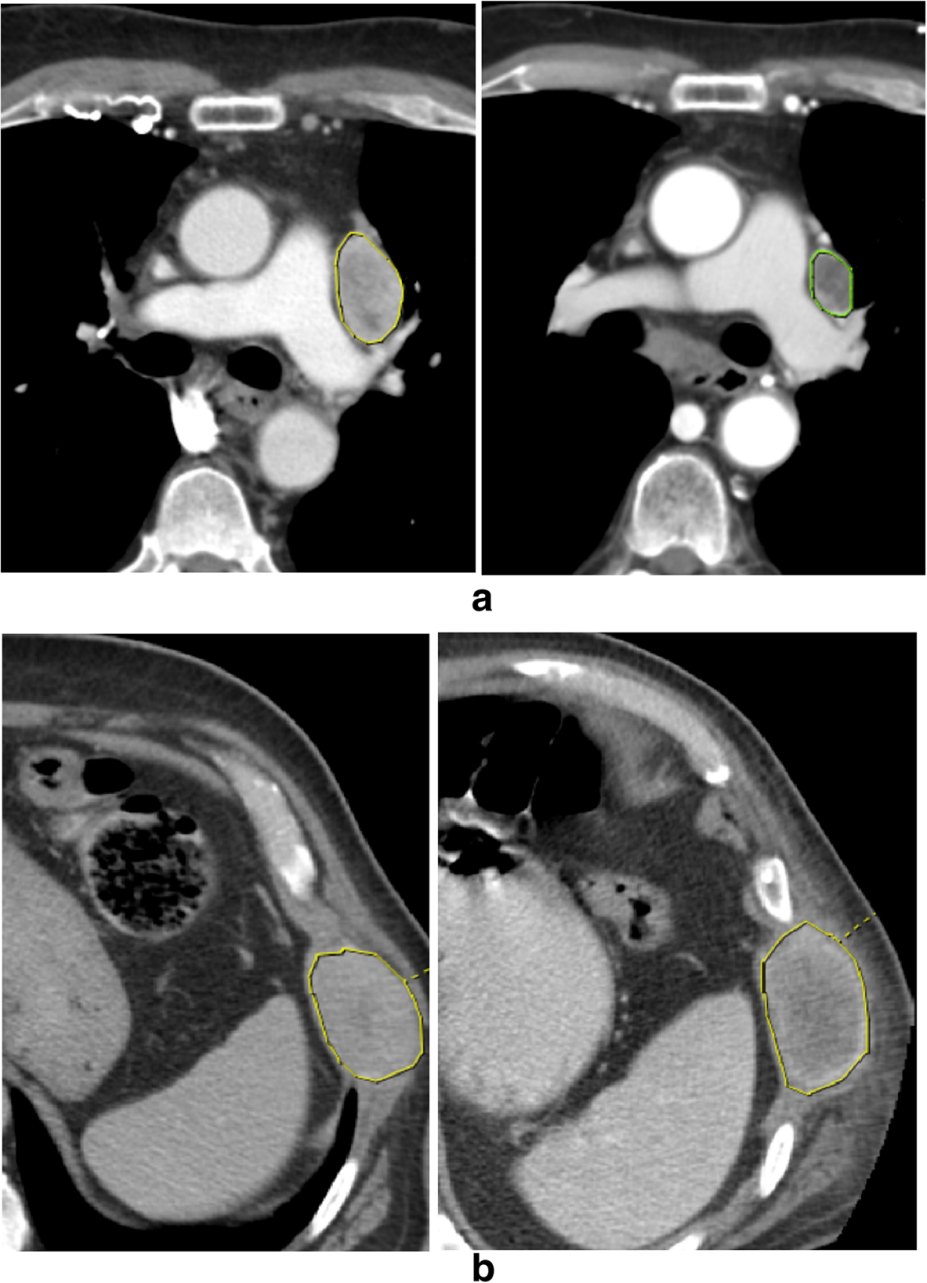
Baseline CT in patients with metastatic clear cell renal cell carcinoma and intermediate IMDC score (Left: prior to treatment, Right: following the treatment). **a** 72-year-old man: OS and PFS in this patient was 1074 and 1063 days, respectively with a baseline nSD = 0.66. Although the tumor remains heterogeneous in appearance there is response by RECIST criteria. **b** 40-year-old man: OS and PFS in this patient were poor at 159 and 113 days, respectively with a lower baseline nSD of 0.54. The tumor is slightly larger but stable by RECIST criteria. The list of significant parameters for 2 cases is shown in Table 6 below. As it can be seen from the table, higher nSD prior to and following treatment and higher percent change in lesion size (i.e., higher size reduction) were associated with higher survival; and higher entropy following treatment and entropy change predicted decreased survival. It should be noted that for PFS, only nSD prior to and following treatment were statistically significant parameters

**Fig. 2:**
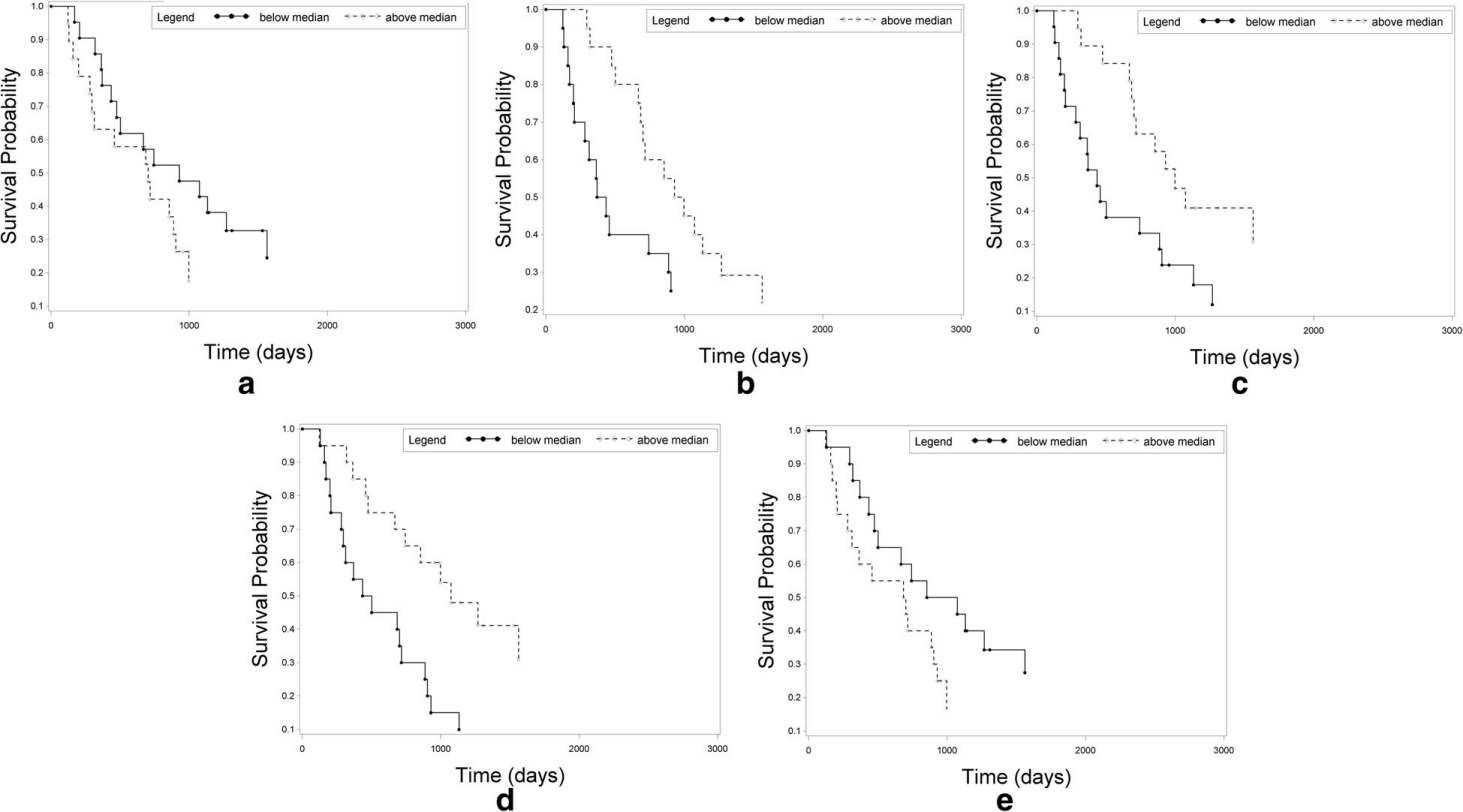
Kaplan-Meier plot of cumulative overall survival (OS) (**a**) Entropy following treatment (median: 4.76), (**b**) nSD prior to treatment (median: 0.58), (**c**) nSD following treatment (median: 0.56), (**d**) Percent change in size (median: 7.65), and E) Entropy Change (median: −0.036)

**Fig. 3:**
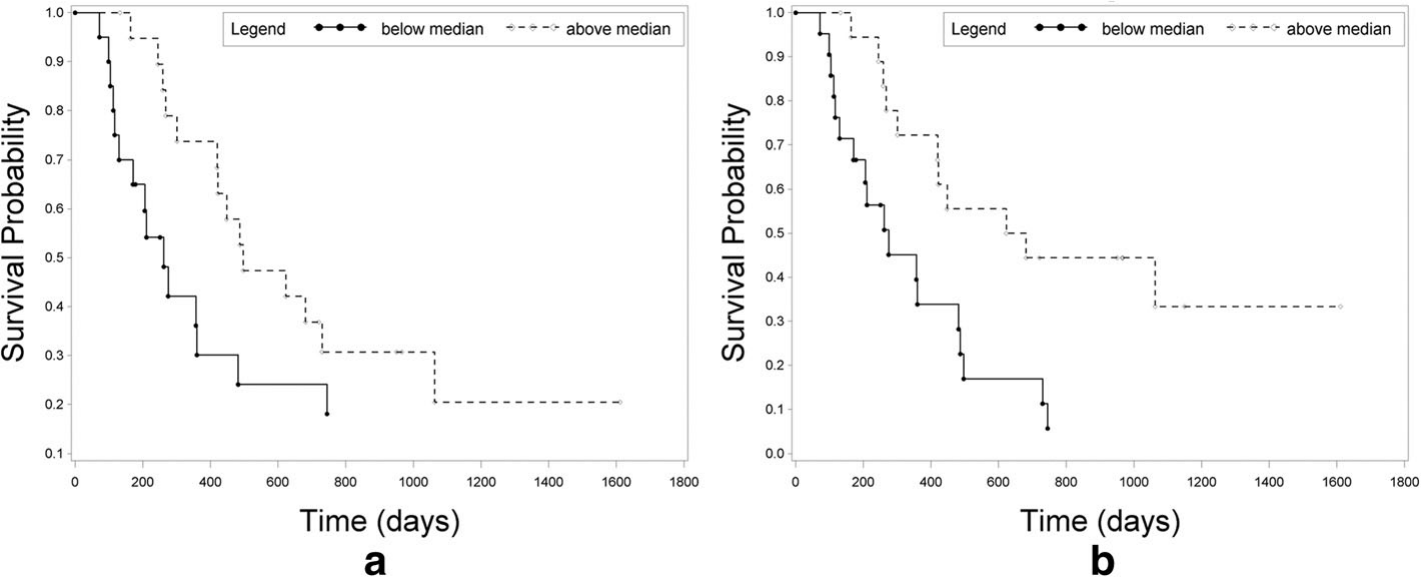
Kaplan-Meier plot of cumulative progression free survival (PFS) (**a**) nSD prior to treatment (median: 0.58) and (**b**) nSD following treatment (median: 0.56)

Examples of CT images demonstrating the appearance of lesions in patients with varying outcomes (OS and PFS) are presented in Fig. 1 and Table 6. The Kaplan-Meier plots for significant parameters for OS and PFS are shown in Figs. 2 and 3.

**Table 6:** The list of significant parameters (with median values) for 2 cases shown in Fig. 1

	OS	PFS	Entropy following treatment	nSD prior to treatment	nSD following treatment	Percent change in size	Entropy Change
Median	729	358	4.76	0.58	0.56	7.65	−0.036
A	1074	1063	4.57	0.66	0.88	33	−0.119
B	159	113	4.79	0.54	0.55	−1	−0.002

### Discussion

In this study, we demonstrate that texture related CT QIB’s are predictive of OS and PFS in metastatic RCC treated with Sunitinib. This study is different from prior studies including the ones done by Goh et al. [10] and Lubner et al. [11] in that the population is limited to patients with clear cell RCC and that it assesses the imaging features in combination with the IMDC prognostic model for RCC patients treated with targeted therapy [1]. This is clinically relevant as one of the proposed uses of QIB’s for personalized medicine is the selection of optimal drug therapies. In the context of clear cell RCC, there are more than one potential first line TKI’s. Thus, it is important to know the baseline characteristic in the context of single histologic tumor type with the standard first line agent as a monotherapy. We have shown that size normalized standard deviation at baseline and follow-up, tumor size change, entropy at initial follow-up, and entropy change before and after treatment are potentially useful QIB’s.

In addition, the study in Goh et al. [10] only looks at PFS while in this paper, both OS and PFS are studied. Furthermore, we limited our cohort to clear cell carcinoma only and a single TKI drug while Goh et al. [10] includes multiple TKI’s and multiple tumor types. Thus, the cohort used in this study would be more reflective to be used as a benchmark of baseline Sunitinib activity to compare new TKI’s in drug trials. The study by Lubner et al. [11] includes a variety of RCC subtypes and does not specify what drugs used for patient treatment and focuses more on prediction of histologic features.

### CT texture features and IMDS

Given that the IMDC prognostic model is well established in RCC patients, we have demonstrated the potential additional value of nSD to further improve prediction of OS and PFS when added to IMDC. In our study, nSD measured in CT images acquired before and after treatment were added to IMDC model. The entropy change prior to and following the treatment and percentage change in the sum of the largest diameter of the lesions size prior to and post treatment did not make a significant improvement to IMDC in the prediction of OS. In a model comparing size and nSD prior to treatment, the latter was a better predictor of OS.

### CT texture features and RECIST criteria

The fact that early size change measured by RECIST is correlated with OS but not PFS is interesting. This discrepancy might be explained by the fact that PFS is reflective of the time course of a single drug as patients would not switch to a second line TKI unless they progressed while OS includes a course of therapy which may include second line and third line TKI’s. Thus, the early size change on Sunitinib could be reflective of overall responsiveness to the TKI’s family including second and third line therapies. RECIST is a very intuitive QIB. In contrast, the visual correlate of nSD may not be intuitive and this may be a barrier to adoption. Therefore, further study is required to investigate the visual patterns of QIBs such as nSD and their histologic correlates to make them more meaningful to the clinicians so that they are not considered as the output of a black box. A preliminary study has been done in this regard by Miles et al. [18] however, further work is required. For example, it is possible that nSD, by combining the number of voxels in a ROI and standard deviation, is combining both size and variation into a single biomarker and thus, providing value by not wholly dispensing with size as a QIB.

Incorporation of new imaging response criteria such as changes in attenuation, morphology and structure into new classifications (Choi, modified Choi and Morphology, Attenuation, Size, and Structure (MASS) criteria) [4, 8] to assess response to antiangiogenic therapies provides more accurate assessment of tumor response to the targeted therapies, but has some limitations. These criteria require follow-up imaging and are not in themselves QIB’s, however, they are intuitive and pragmatic. Further research into the optimal approach and use of these measures in personalized medicine is required.

### Study limitations

Our study has limitations. In particular, we note that the small sample size did not allow for multiple testing correction for the large number of QIB’s tested. This means that the results for this study remain hypothesis generating and further prospective validation will be required as with other similar studies published to date. In addition, this study was retrospective, raising potential selection biases. For example, patients who had a decline in renal function and did not have contrast on their follow-up were excluded from this analysis.

### Clinical value

Of clinical interest is the value of baseline nSD both as a predictive and prognostic parameter and its potential additive value to the IMDC score at baseline. This opens the door to prospective validation with the aim to define a predictive biomarker for optimal drug selection initially without having to wait for a follow-up scan using a practical and low cost standard of care test, a simple contrast enhanced CT.

## Conclusion

In conclusion, size normalized standard deviation is a quantitative imaging biomarker texture feature that in patients with metastatic clear cell carcinoma may add to the IMDC score in prediction of therapy response and overall survival. Further validation of this biomarker in prospective trials is required.

## Abbreviations

CI: Confidence interval
HR: Hazard ratio
IMDC: International Metastatic Renal-Cell Carcinoma Database Consortium Model
nSD: normalized standard deviation
OS: Overall survival
PerC: Percentage change
PFS: Progression free survival
QIB: Quantitative imaging biomarker
RCC: Renal cell carcinoma
ROI: Region of interest
SD: Standard deviation
TKI: Multi-targeted tyrosine kinase inhibitor
